# The Effect of N-Terminal Domain Removal towards the Biochemical and Structural Features of a Thermotolerant Lipase from an Antarctic *Pseudomonas* sp. Strain AMS3

**DOI:** 10.3390/ijms19020560

**Published:** 2018-02-13

**Authors:** Wahhida Latip, Raja Noor Zaliha Raja Abd Rahman, Adam Thean Chor Leow, Fairolniza Mohd Shariff, Nor Hafizah Ahmad Kamarudin, Mohd Shukuri Mohamad Ali

**Affiliations:** 1 Enzyme and Microbial Technology Research Center, Universiti Putra Malaysia, Serdang 43400, Selangor, Malaysia; wahhidalatip@gmail.com (W.L.); hafizah_kamar@upm.edu.my (N.H.A.K.); 2 Enzyme and Microbial Technology Research Center, Department of Microbiology, Universiti Putra Malaysia, Serdang 43400, Selangor, Malaysia; fairolniza@upm.edu.my; 3 Enzyme and Microbial Technology Research Center, Department of Cell and Molecular Biology, Universiti Putra Malaysia, Serdang 43400, Selangor, Malaysia; adamleow@upm.edu.my; 4 Enzyme and Microbial Technology Research Center, Department of Biochemistry, Universiti Putra Malaysia, Serdang 43400, Selangor, Malaysia; mshukuri@upm.edu.my

**Keywords:** microbial enzyme, N-terminal domain, biochemical characterization, in silico, lipase, antarctic

## Abstract

Lipase plays an important role in industrial and biotechnological applications. Lipases have been subject to modification at the N and C terminals, allowing better understanding of lipase stability and the discovery of novel properties. A thermotolerant lipase has been isolated from Antarctic Pseudomonas sp. The purified Antarctic AMS3 lipase (native) was found to be stable across a broad range of temperatures and pH levels. The lipase has a partial Glutathione-*S*-transferase type C (GST-C) domain at the N-terminal not found in other lipases. To understand the influence of N-terminal GST-C domain on the biochemical and structural features of the native lipase, the deletion of the GST-C domain was carried out. The truncated protein was successfully expressed in *E. coli* BL21(DE3). The molecular weight of truncated AMS3 lipase was approximately ~45 kDa. The number of truncated AMS3 lipase purification folds was higher than native lipase. Various mono and divalent metal ions increased the activity of the AMS3 lipase. The truncated AMS3 lipase demonstrated a similarly broad temperature range, with the pH profile exhibiting higher activity under alkaline conditions. The purified lipase showed a substrate preference for a long carbon chain substrate. In addition, the enzyme activity in organic solvents was enhanced, especially for toluene, Dimethylsulfoxide (DMSO), chloroform and xylene. Molecular simulation revealed that the truncated lipase had increased structural compactness and rigidity as compared to native lipase. Removal of the N terminal GST-C generally improved the lipase biochemical characteristics. This enzyme may be utilized for industrial purposes.

## 1. Introduction

The flexibility of microbial lipases in terms of temperature, pH levels and organic solvent action has attracted industry attention [1]. Lipase are ubiquitous and have a strong market share in biocatalysts, with extensive usage in the detergent and food industries [2]. Many microbial lipase genes have been cloned and commercialized in the past several years from microorganisms including *Candida rugosa* (formerly C. cylindracea), *Rhizomucor miehei*, *Rhizopus delemar*, *Geotrichum candidum*, *Burkholderia cepacia* (formerly *Pseudomonas*), *Pseudomonas pseudoalcaligenes*, *Pseudomonas mendo-cina* (originally *Pseudomonas putida*) and *Chromobacterium glumae*. The majority of these enzymes have proven industrial applications [3]. Lipases originating from *Pseudomonas* are potential industrial enzymes. Lipases from the *Pseudomonas* family have contributed to general understanding of lipase activity and function. *Pseudomonas* lipases have been reported to be two-domain enzymes, with some with RTX (repeat in toxin) located in the C-terminal domain [4] and some active in the presence of chaperone located in the N-terminal domain. [5]. 

The function of the proteins relies on global (fold) or local similarities [6]. The well-known low stability of cold-adapted enzymes and the postulated involvement of molecular plasticity or flexibility in cold activity [7] have spurred analysis of structural factors responsible for stability using available X-ray structures. The flexibility of the enzyme is affected by factors including active site, surface area, loop and the stability of both lid region and secondary structure [8]. The N and C terminals have been reported to be the most flexible regions in the protein structure, playing important roles in protein folding, native state stability and final turnover [9]. The deletion of 42 amino acids in the N-terminal domain of cold adapted lipase from *Psychrobacter cryohalolentis* K5T increases thermostability. However, the optimum temperature was reduced by 5 °C (25 to 20 °C) [10]. This is in contrast with lipase from *Pseudomonas protegens* Pf-5, as that enzyme lost catalytic activity when the N-terminal domain was removed [11]. The modification of a flexible region such as the N and C-terminals may affect the stability and flexibility of the protein. These changes will result in changes in biological functions and biochemical properties. [12]. 

In a previous study, AMS3 lipase was cloned and over expressed in *E. coli* expression system [13]. AMS3 lipase was able to undergo hydrolysis at broader temperature and pH ranges. Long chain triglyceride and natural oils are preferable. The enzyme shows potential in biodiesel synthesis as it is able to tolerate several hydrophobic and hydrophilic organic solvents. In this study, we remove the N-terminal flexible domain, of AMS3 lipase. The truncated AMS3 lipase activity and predicted structure are characterized and summarized in this work. 

## 2. Results and Discussion

### 2.1. Homology Modelling and Molecular Dynamic Simulation of AMS3 Lipase

**Figure 1:**
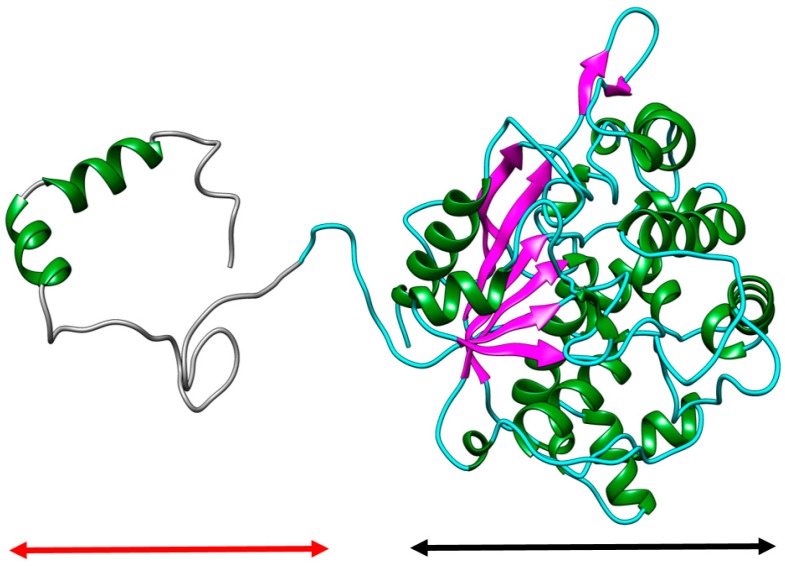
The preliminary structure of AMS3 lipase model built with Raptor X. The secondary structure indicated by the red arrow shows GST C *Pseudomonas* sp. form in α helix and coils. The lipase domain is indicated by black arrow. The GST C *Pseudomonas* sp. contains 57 amino acid. Magenta: β-sheet; Green: α-helix; Cyan: coil; Grey: GST-C *Pseudomonas* sp. AMS3.

A native model was built using Raptor X software (raptorx.uchicago.edu) and evaluated to ensure correctness of the model stereochemistry, as checked by a Ramachandran plot [14]. Verify 3D and ERRAT are other common tools used to verify protein structure. The Ramachandran plot scores of the predicted structure of AMS3 lipase showed that 96.7% of the amino acid was in a favorable region, with 2.9% in the allowed region and only 0.2% in disallowed region. Proline and Phenyalanine contribute more to the disallowed region. The ERRAT score of predicted AMS3 lipase structure showed 91.17% quality, with a calculated error value of less than 99%. A few amino acids at 41, 57, 58 and 401 contributed to the 99% error value. However, the overall structure can be considered a good quality structure at higher than 90%, as shown by ERRAT. The accuracy of such a model can also be evaluated by Verify 3D, which determines the source of a protein structure such as X-ray, NMR and computational process. According to the Verify 3D profile, the native sequences were compatible with their 3D structure validation. The 3D-1D structure score was 89.56% evaluated over the 0.2. The structure at the N-terminal contributed to the low structure value. The overall validation results show that the structure is suitable for further experimentation (Molecular Dynamic simulation). The overall predicted native structure contains two domains, namely the GST C domain located at the N-terminal and the lipase domain at the C-terminal. The predicted native structure contains 22 α-helixes and 9 β-sheets commonly found in α/β hydrolase group. A preliminary model of the lipase is shown in Figure 1. As previously reported, the predicted catalytic triad using sequence alignment Ser170, Asp364 and His406 are in a catalytic configuration buried in the protein core. Many cold adapted lipase structures have been reported to contain a flexible region of α-helix and coil like the secondary structure of GST C *Pseudomonas* sp. Glutathione *S*-transferase (GST) from *Pseudoalteromonas* sp. ANT506 was reported to play an important role in temperature adaptation, other than that it was also reported to be able to adapt a broader pH condition in *Pseudomonas* sp. DJ77 and *E. coli* B [15]. The α-helices and coils are known to be more flexible than β-sheets. These flexible regions affect the stability of cold-adapted lipase structures as compared to thermophilic ones. The increased loop conformation was thought to be one of the factors responsible for enzymes activity at low temperature [16]. The results of this study support the CD that the secondary structure of the native has many α-helices and coils. The removal of these regions at the N-terminal region might increase the protein stability and rigidity of the AMS3 lipase. Lipases from *Staphylococcus epidermidis* AT2 [17] and *Psychrobacter cryohalolentis* K5 [10] became more stable and rigid without the flexible region.

**Figure 2:**
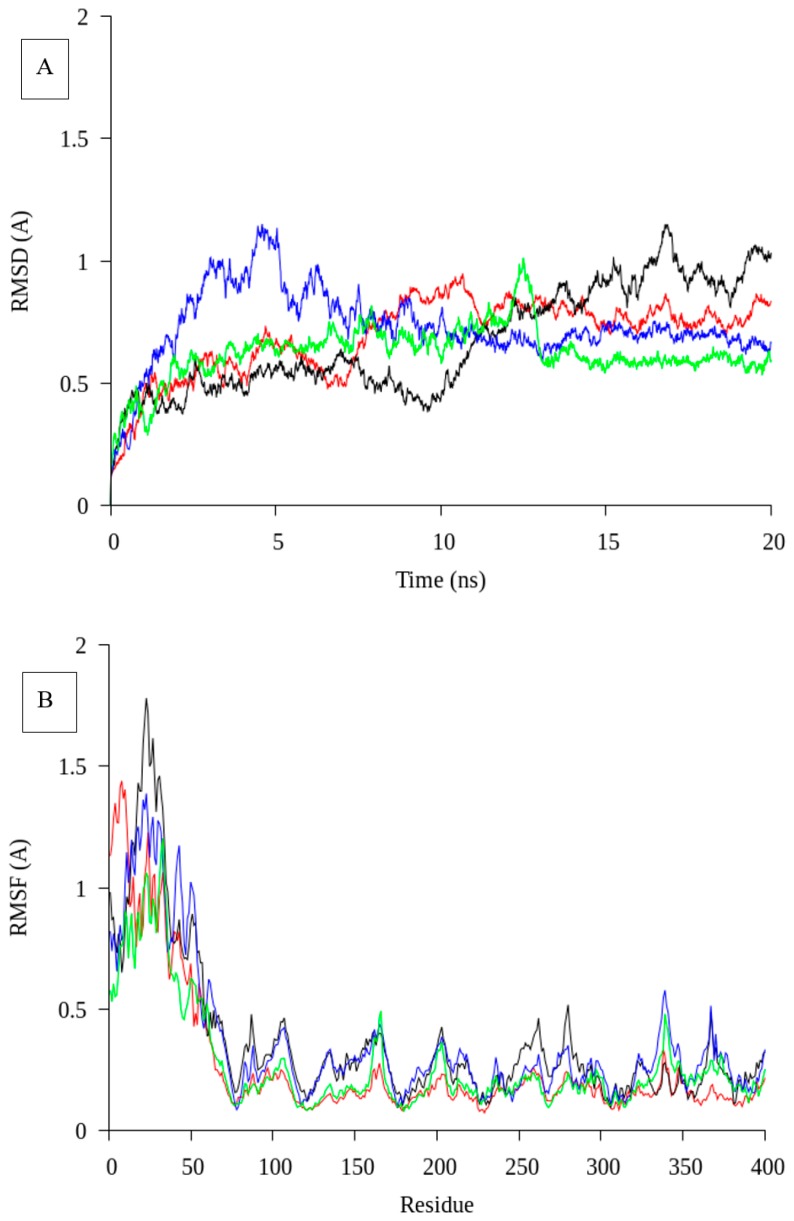
Molecular dynamic simulation of native at varies temperature. (**A**): RMSD value for AMS3 lipase in 20 ns; (**B**): RMSF value for AMS3 lipase in 20 ns. The red line represents a temperature of 10 °C; the Black line was 20 °C; Green was 30 °C; and Blue was 40 °C.

A molecular dynamic simulation was carried out using GROMACS version 4.0.5 [18] to determine the molecular movements of the lipase at different temperatures. The temperatures were set from 10 °C till 40 °C as the native was reported stable this temperature range [13]. Conformational changes in protein flexibility and stability were determined by root-mean-square deviation (RMSD) for the entire structure or molecule and root-mean-square fluctuation (RMSF) for each residue, respectively. The RMSD value was calculated after 10 ns in order to equilibrate the system. The RMSD of native was analyzed from protein backbone atom in 20 ns (Figure 2A). The highest RMSD value was determined for native at 17 ns, which is more than 1 Å at 20 °C. The 20 °C structure did not show a plateau line in the graph during the trajectory. This result might contribute to the lower stability of the lipase. In contrast, at 30 °C a plateaued trajectory was visible from 10 ns until the end. The RMSD value of MD at 30 °C was lower than others temperature at 0.5 Å. The lipase activity from the previous report showed that native was very stable at 30 °C [13]. The MD simulation was found to complement the experimental results reported before.

The flexibility of the protein structure was determined using RMSF as calculated from C_α_ (Figure 2B). Flexible region at N- and C-terminal are often observed in cold adapted structure. Similar to AMS3 lipase (native), the N-terminal domain (GST-C *Pseudomonas* sp. A3) showed higher movement (fluctuation) at 20 ns of trajectory in all temperatures. Fluctuation of N-terminal domain might affect the protein structure and could contribute to high mosaicity. RMSD and RMSF are related to protein stability and flexibility. The stability of a protein is an important factor which enhances its biomolecular functions, activity and protein regulation [19]. Protein aggregation and misfolding occur when a protein structure loses thermodynamic stability [20]. These factors might contribute to high protein mobility or flexibility and promote larger assembly of proteins, further leading to aggregation. For comparison purposes, the full-length AMS3 lipase is designated native and the modified enzyme is designated truncated AMS3 lipase.

**Figure 3:**
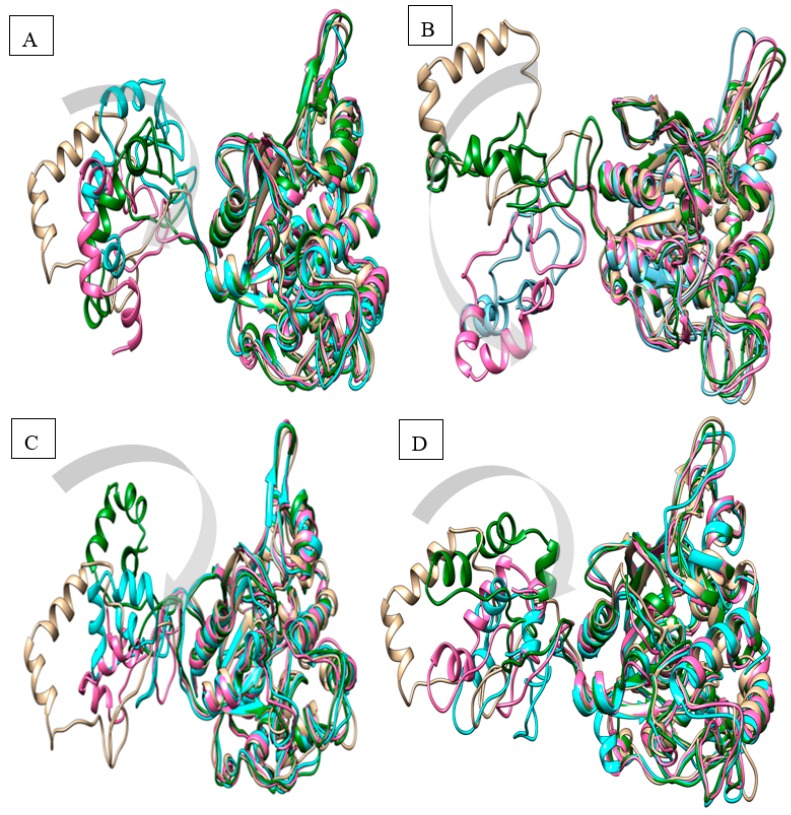
Superimposition of the simulated native lipase at different time intervals. (**A**): 10 °C; (**B**): 20 °C; (**C**): 30 °C; (**D**): 40 °C. Native: brown (0 ns); Green: 10 ns; Pink: 15 ns; Cyan: 20 ns. Movement of the N-terminal domain is indicated by a grey arrow.

To visualize the structural changes, a superposition of the whole native structure was performed between 0 and 20 ns (Figure 3). The movement of N-terminal domain was calculated using Chimera software (www.cgl.ucsf.edu/chimera). This is because the N-terminal domain mostly contributes to fluctuation and increases the flexibility of a protein. The N-terminal structure was observed to be constantly mobile. Distances were calculated from the structure template at 5 ns intervals from 0 ns till 20 ns. The distances measured from 0 ns till 20 ns are depicted in Table 1. This flexible region with larger fluctuation would trigger an unfolding process and the same time will lead to denatured protein [21]. These results show that the N-terminal contributed to the flexibility of the protein, leading to protein aggregation [22,23]. Alternatively, to improve the prospects of getting a rigid and less flexible structure, the deletion or truncation of the flexible region (GST-C) would be a promising strategy.

#### Homology Modelling and Molecular Dynamic Simulation of Truncated AMS3 Lipase

**Figure 4:**
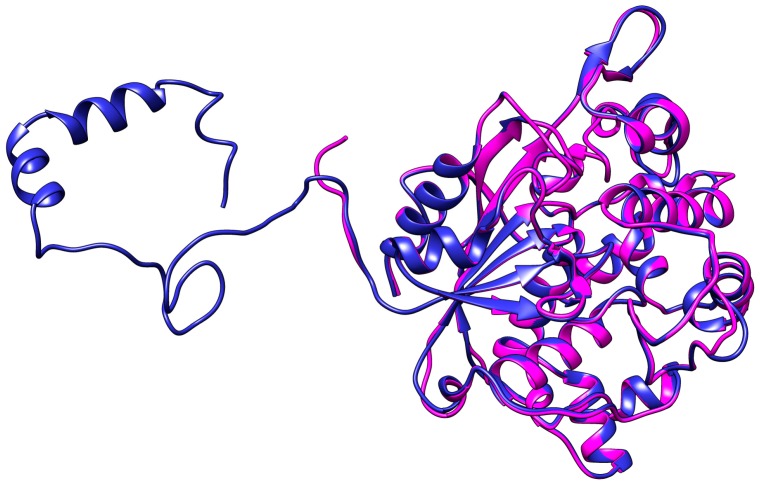
Superimposition of AMS3 lipase (native) and truncated AMS3 lipase. Blue represents the native and magenta the truncated AMS3 lipase. The RMSD value for lipase domain was 0.35 Å.

**Figure 5:**
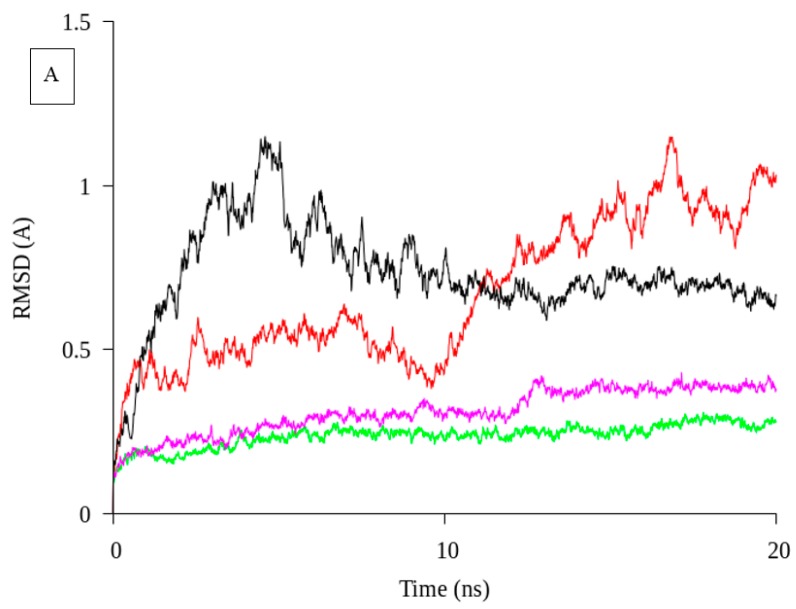

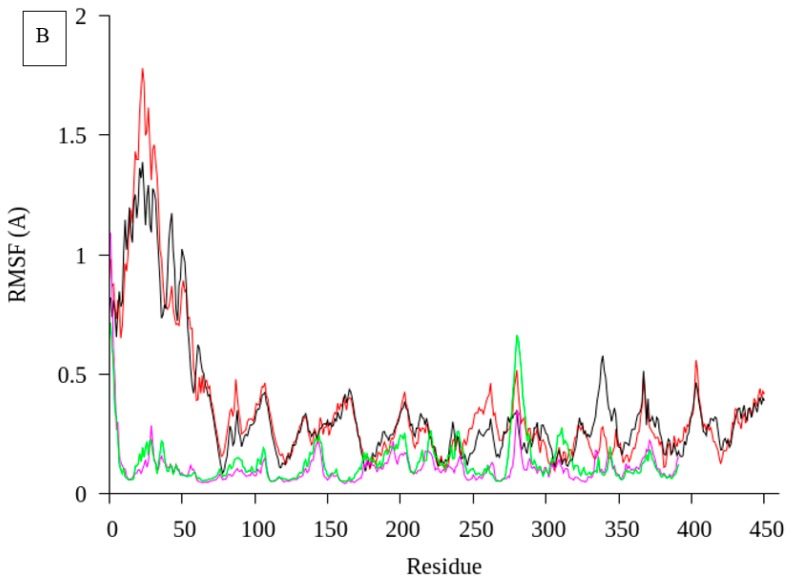
Molecular dynamic simulation of truncated AMS3 lipase at 20 and 30 °C. (**A**): RMSD value for truncated AMS3 lipase in 20 ns; (**B**): RMSF value for truncated AMS3 lipase in 20 ns. Color in Red: native in 20 °C; Black: native 30 °C; Green: Truncated AMS3 lipase in 20 °C; Magenta: Truncated in 30 °C.

In order to support the data that deletion of N-terminal domain affected the biochemical characterization, in silico analysis was attempt. The predicted structure of truncated AMS3 lipase was built by the deletion of N-terminal domain (GST-C), contributing to the flexibility of the protein. The predicted model was built using the same free web software. The predicted structure was also evaluated using the same validation software packages, including Ramachandran plot, Verify 3D and ERRAT. Validation results show that the structure was a good 3D structure based on the score which was 100%. Predicted structure of truncated AMS3 lipase shown a better score compare with native, where all validation parameter showed more than 90% score. The predicted structure of truncated AMS3 lipase was then started for MD simulation. Predicted structure of truncated AMS3 lipase consist of 18 α-helix and 9 β-sheet. In contrast, the native predicted structure contains higher α-helix and coil content. Random or coil structure has been reported to make a protein less rigid or flexible [24]. The superposition of these two structures is shown in Figure 4. The RMSD value was 0.35 Å (lipase domain). No significant changes were observed. To gain structural insight into in silico analysis, molecular dynamic simulation (MD) was attempted to predicted model of truncated AMS3 lipase. The simulation was performed parallel with the native to determine the differences between native and truncated AMS3 lipase. As shown in Figure 5, the RMSD value for truncated AMS3 lipase was lower than native in both temperatures. The values were below 0.5 Å. During the trajectory of truncated AMS3 lipase (without the N-terminal), a plateaued line was visible as early as 5 ns till end. A longer trajectory ensures investigation over well-thermalized systems [25]. Alizadeh-Rahrovi et al. also reported a lower value of RMSD contributed to structural stability [26]. The influence of terminal moiety to the enzyme stability also been study by the L1 lipase using the same method (MD). The experiment showed that the N-terminal domain, as well as the small domain, play an important role in protein stability toward high temperature. MD was measured at varying temperature such as 300, 400 and 500 K in order to show the fluctuation of the N-terminal domain [27]. 

To attain information of local structural flexibility and determine the differences between the native and truncated structure, an RMSF study was attempted for these two predicted structures for each amino acid at temperatures of 20 and 30 °C. Temperature selection was based on biochemical characterization of truncated AMS3 lipase. A few amino acids at N-terminal contributed to fluctuations in the RMSF graph for truncated AMS3 lipase but the values were still lower than those of the native. The terminal regions (N- or C-terminal) are considered more flexible than other parts of the proteins [28]. MD simulation reveals that flexible regions contribute less to the thermal stability of a protein.

### 2.2. Modification of AMS3 Lipase

#### Deletion of N-Terminal Domain

A sub-cloning strategy was used to clone the truncated AMS3 lipase into pET 51b, after which the GST C Pseudomonas at N-terminal of the enzyme was deleted (57 amino acids). In this strategy, the truncated AMS3 lipase was amplified using standard PCR protocol. The vector was linearized using Kpn1 and Sac1 restriction enzymes prior to the ligation of the PCR products. The ligation product was used to transform *E. coli* Top10 and express the protein in the *E. coli* BL21 (De3) host. The recombinant *E. coli* harboring the truncated AMS3 lipase gene exhibited lipolytic activity when plated on the tributyrin agar plate. The removal of the N-terminal domain did not affect the hydrolytic activity of the lipase in general. The enzyme was folded properly, even without the N-terminal domain, as the recombinant truncated lipase was able to perform hydrolysis reactions. In contrast, S5 lipase from *Pseudomonas aeruginosa* was reported as chaperone-dependent lipase [5].

### 2.3. Purification of Truncated AMS3 Lipase

**Figure 6:**
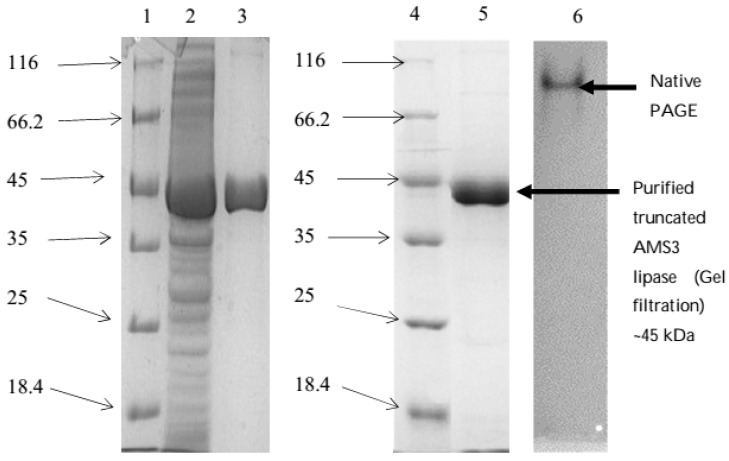
Polyacrylamide gel electrophoresis (PAGE) profile of the recombinant truncated AMS3 lipase. Lane (1) Unstained protein molecular weight marker (Fermentas, Germany); Lane (2) Crude truncated AMS3 lipase; Lane (3) Truncated AMS3 lipase purified through affinity chromatography; Lane (4) Unstained protein molecular weight marker; Lane (5) Purified truncated AMS3 lipase; Lane (6) Native-PAGE.

The truncated AMS3 lipase was purified using the same affinity chromatography (nickel sepharose) method as native. Contrary to native, a Tris-HCl buffer at a pH of 8 was found to be suitable buffer for purification. However, all other purification parameters (steps) remained the same as the native. In contrast to native, the truncated AMS3 lipase was subjected to a second step of purification to increase protein purity. Determination of the protein molecular weight and homogeneity was performed by SDS-PAGE and Native-PAGE, respectively (Figure 6).

A summary of purification table is shown in Table 2. Recovery of the truncated AMS3 lipase was higher than the 37% of the native at 87%. Purification was successful even after the second purification, with a recovery rate of 65.4%. After the first and second steps of purification, the purified lipase with 5× and 7× folds were compared with crude. *Pseudomonas* sp. Strain B11-1 lipase showed a similar trend of recovery as the truncated AMS3 lipase, giving a higher enzyme fold after the second step of purification [29]. The active fractions were pooled together and determined the protein molecular weight and homogeneity by SDS-PAGE and Native-PAGE, respectively (Figure 6), while 8 µg/mL of the protein was loaded onto the gel. The expected size of the truncated AMS3 lipase, including fusion tags, was around ~45 kDa. 

### 2.4. Biochemical Characteristics of Truncated AMS3 Lipase

#### 2.4.1. Effect of Temperature

**Figure 7:**
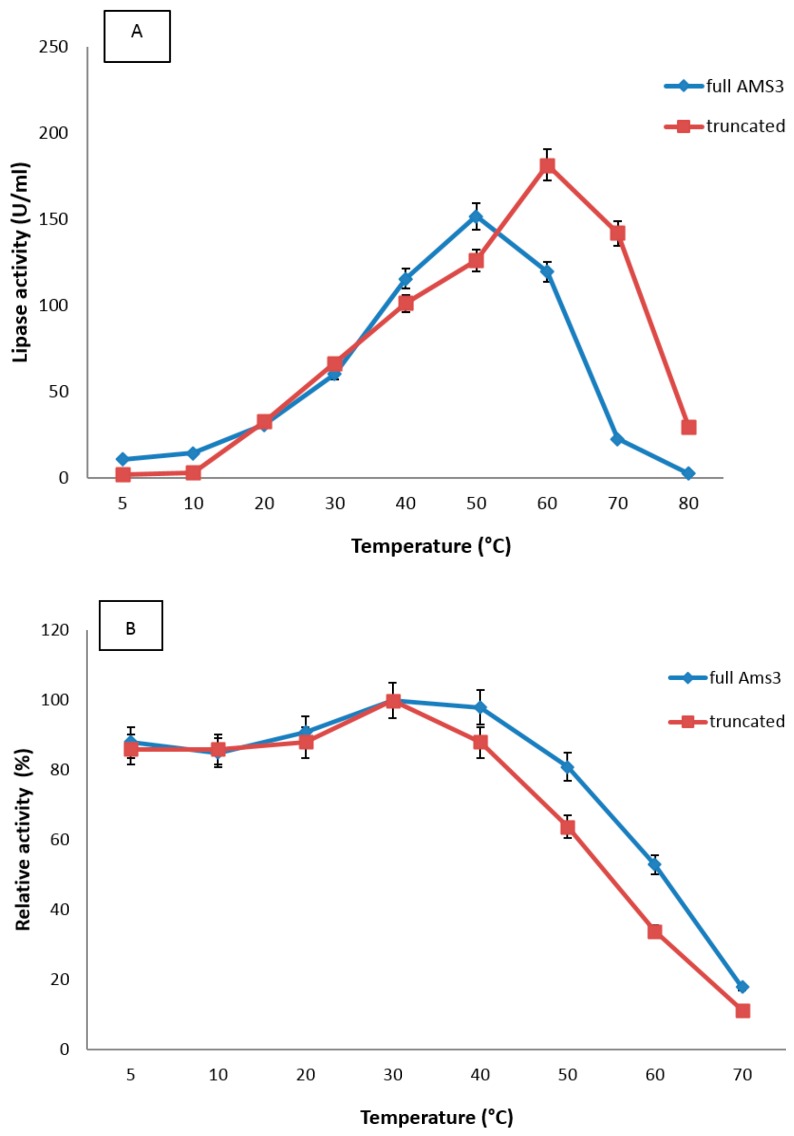
Effect of temperature on purified truncated AMS3 lipase activity. The lipase assay was performed with olive oil as a substrate. (**A**) Enzyme samples were assayed at various temperatures. The temperature that showed the highest lipase activity was considered to be the optimum temperature for the truncated AMS3 lipase; (**B**) Enzyme stability was expressed as a percentage of the control. The enzyme was incubated at various temperatures for 30 min. Assays were performed at optimum temperatures Relative activity was calculated with 30 °C as 100%. Red line: Truncated AMS3 lipase; Blue line: AMS3 lipase. Significant statistical differences according to ANOVA (*p* < 0.05).

Lipase activity was measured under temperatures ranging from 5 to 70 °C. The truncated AMS3 lipase exhibited am optimum temperature 10 degree higher (60 °C) than the native lipase (Figure 7). Deletion of the N-terminal domain made the structure less flexible and more rigid, whereas the GST-C protein was reported to be able to tolerate with temperature [15], contributing to the shift of thermostability profile. The truncated AMS3 lipase was still able to retain some native features, displaying stability at broader temperatures with a relative activity of over 60% at 5 to 40 °C before markedly declining at temperatures above 40 °C. *Pseudoalteromonas lipolytica* SCSIO 04301, *Candida antarctica* ZJB09193 and *Pseudomonas* sp. strain B11-1 were isolated from a cold environment and found to produce thermotolerant lipases. These lipases showed maximum activity at 45, 52 and 45 °C, respectively [29,30,31]. These lipases were reported to be stable at low temperature range of 10–40 °C but sharply decreased in their activity at higher temperatures. In contrast, the *Oceanobacillus* strain PT-11 lipase showed maximum activity at 10 °C. The lipase has been reported to be cold-adapted, even though the bacterium was isolated and grown at 37 °C [32]. This lipase also showed a similar pattern of activity to native and truncated AMS3 lipases, remaining stable in a temperature range of 5–40 °C. 

#### 2.4.2. Effect of pH

**Figure 8:**
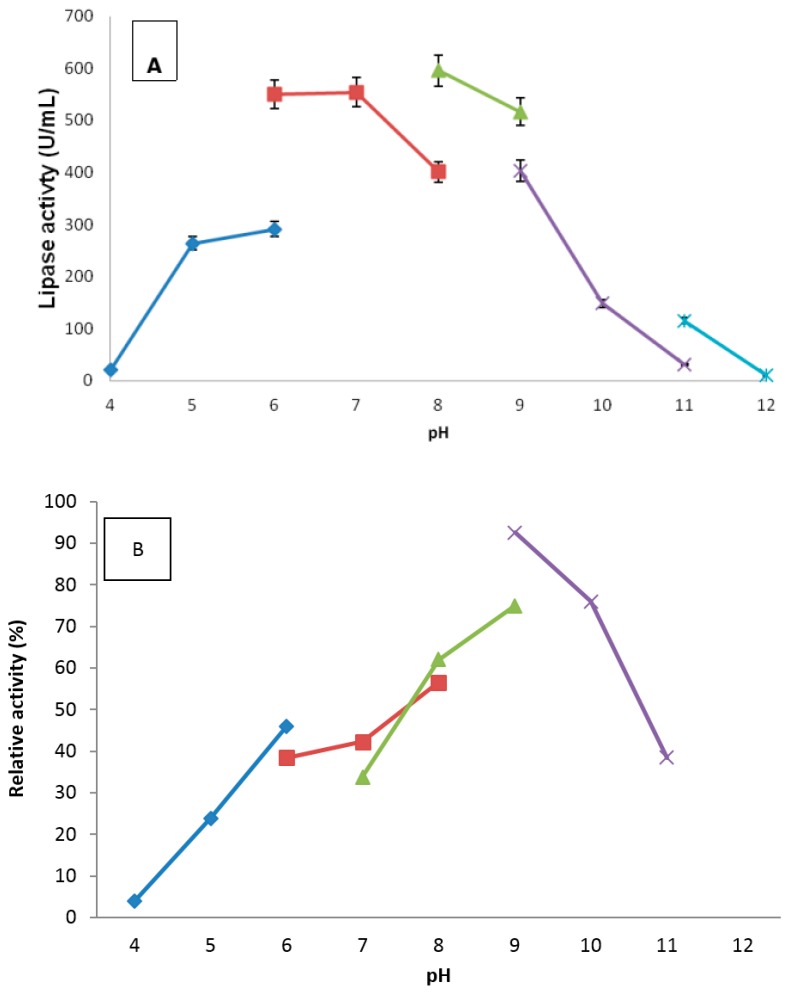
Effect of pH on purified truncated AMS3 lipase activity (**A**); The lipase activity was measured at different pH using olive oil emulsion as substrate. Effect of the pH on purified truncated AMS3 lipase stability is shown in (**B**). The enzyme was pre-incubated in different buffers for 30 min. Lipase assays were performed at 60 °C. Sodium acetate buffer was used for the pH range of 4.0 to 6.0 (♦); potassium phosphate buffer for the pH range of 6.0 to 8.0 (■); Tris-HCl buffer for the pH range of 8.0 to 9.0 (▲); and glycine-NaOH buffer for the pH range of 9.0 to 12.0 (x). Significant statistical differences according to ANOVA (*p* < 0.05).

The truncated AMS3 lipase showed optimum activity at pH 8.0 (Figure 8), as observed with the native. The truncated AMS3 lipase demonstrated stability only at pH 8–10 (Figure 8B). The removal of a few residues might have been the reason for pH profile shifts of the truncated AMS3 lipase to more alkaline condition (alkaline enzyme). A pI value of 6.2 was calculated for the truncated AMS3 lipase. The native enzyme pH profile was more active at pH 5–10 condition. Ogino et al. reported a lipase from *Pseudomonas aeruginosa* LST-03 with broader pH stability at pH 5–8. Two additional lipases were reported by the same authors, namely lip3 and lip8, share pH stability with the Native AMS3 lipase. However, the lip8 has optimum pH 7 more than the native [33,34,35]. pH stability is a unique feature of most lipases including the *Pseudomonas* lipase. Most *Pseudomonas* lipases, including the native and truncated AMS3 lipases, achieve high activity at alkaline condition. In contrast, *Pseudomonas gessardii* lipase demonstrated optimum activity at acidic pH [36]. Among the 57 amino acids that were deleted, nine carried a positive charge (one arginine, seven lysine and one histidine), while six carried a negative charge (five from aspartic acid and one from glutamates). Each amino acid contributes to pI value of the protein and deleting the 57 amino acids at the N-terminal domain might have changed the isoelectric point of the protein that contributes to pH stability changes. In addition, glutathione *S*-transferase protein (GST-C) isolated from a few organisms has been reported to be able to tolerate a wide range of pH levels [15]. These results are in line with those reported for *Bacillus thermocatenulatus* lipase, which had 20 amino acids deleted from the native. The optimum pH stability shifted more to alkaline conditions compared to the native [37].

#### 2.4.3. Effect of Metal Ions

**Figure 9:**
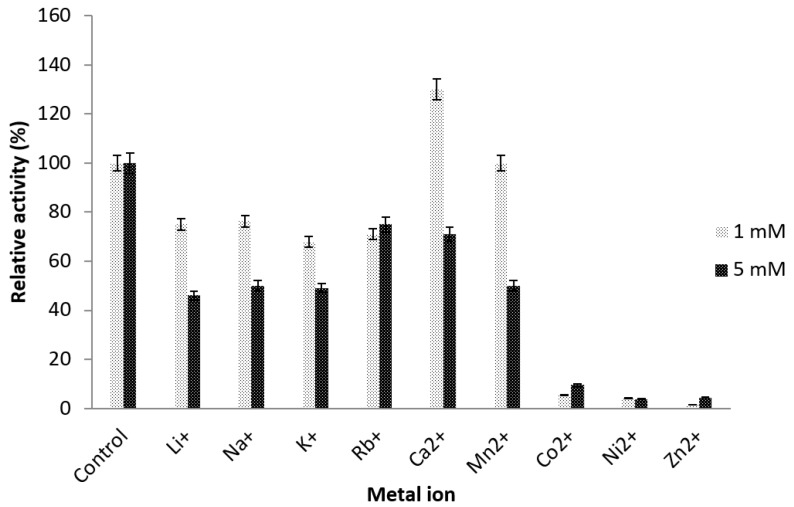
Effects of metal ions on truncated AMS3 lipase stability. Truncated AMS3 lipase activity was enhanced in the presence of a few metal ions. The enzyme was pre-incubated at 60 °C with various metal ions at concentrations of 1 and 5 mM.

Metal ions often serve as stimulants in enzyme reactions. The role of metal ions in enzyme reaction in the presence of a substrate is tightly bound to the lid or active site [38,39]. In some cases, the metal ions enhance the enzyme activity toward the substrate. The effects of metal ions were studied on purified truncated AMS3 lipase. For all the metal ions studied at the concentrations of 1 and 5 mM (Figure 9). The truncated AMS3 lipase showed higher activity of 130, 100 and 76.23 U/mL in the presence of calcium, manganese and sodium respectively. Calcium has been reported to enhance lipase activity from *Candida antarctica* lipase and *Bacillus amyloliquefaciens* from 100% to 151% and 110% respectively. A high concentration of calcium (5 mM) led to a decline in lipase activity to 71%. Similar observation was also recorded to *Staphylococcus epidermidis* AT2 lipase whose activity was declined from 140% to 70% [17]. Similar to the native, the truncated AMS3 lipase remained active even when treated with metal ions such as manganese and lithium at 1 mM concentration. The enzyme was found to be active above 50% of relative activity compared to the untreated one. At higher concentrations, transition metal ions become toxic to proteins leading to their deactivation. Both the truncated AMS3 and native lipases can be classified as metal-activated enzymes.

#### 2.4.4. Specific Activity of the Truncated Lipases to Different Substrates

**Figure 10:**
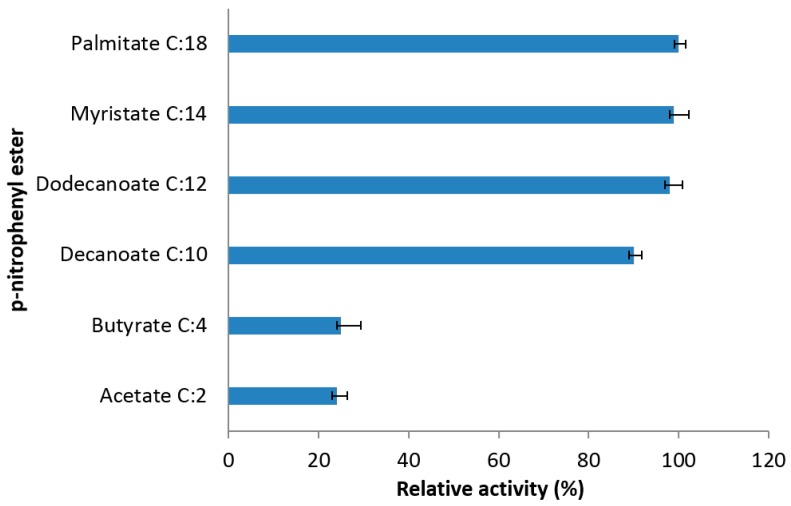
Substrate specificity of purified truncated AMS3 lipase towards *p*-nitrophenyl ester. The data shown are means of triplicate measurements expressed as% activity relative to those of palmitate.

The truncated AMS3 lipase showed preference for long-chain *p*-nitrophenyl esters (Figure 10). Palmitate (C16) was a control and calculated as 100% of relative activity. Ninety percent of relative activity was observed with long-chain *p*-nitrophenyl substrate. This proves that the enzyme is a true lipase which acts at the oil-water interface of water-insoluble long-chain fatty acid esters [40]. The results are comparable to native and lipases from *Pseudomonas* sp. monteilii TKU 009 and *Bacillus coagulans* BTS-3 in terms of the preferred long chain carbon substrates [41,42]. Deletion of the N-terminal domain did not change the substrate specificity of the truncated AMS3 lipase, maintaining the features of a true lipase. Moreover, the deleted residues were far from the lid and the active site.

#### 2.4.5. Effect of Organic Solvents

**Figure 11:**
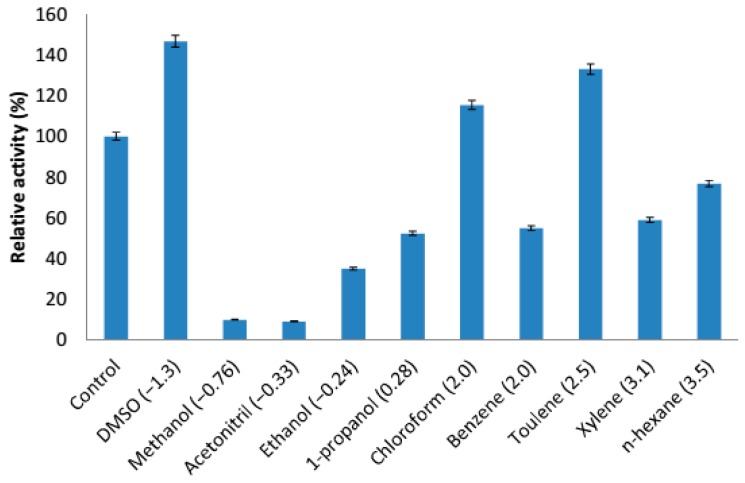
Effect of organic solvents on purified truncated AMS3 lipase. The lipase activity was expressed relative to the control (without solvent). Log *p* (water/octanol coefficient) value of each solvent is presented in parenthesis. 25% *v*/*v* organic solvents were used.

The truncated AMS3 lipase was tested with various organic solvents divided into polar and non-polar based on log *p* value. The majority of solvent tolerant lipases were reported from *Pseudomonas* sp. and *Bacillus* sp. with very limited information on the cold-adapted bacteria [43]. The truncated AMS3 lipase was fairly stable in both polar and non-polar organic solvents (Figure 11). Activation of the enzyme leads to high relative activity and versa for inactivation of activity when treated with organic solvents. The mixtures of enzyme, buffer (50 mM Tris-HCl buffer pH 8) and organic solvent (25% *v*/*v*) were incubated at a temperature of 60 °C. The truncated AMS3 lipase activity was increased by organic polar solvents such as DMSO to about 146.7% compared to the control at a temperature of 60 °C. Polar organic solvents cause more protein instability compared to non-polar ones. Moreover, many polar organic solvents have demonstrated negative effects on enzymes activity. Compared to native, the truncated enzyme was less tolerant (deactivated) to methanol when treated for 1 h. The activity was less than 50% as compared to the untreated enzyme. Additionally, the truncated AMS3 lipase demonstrated high tolerance towards non-polar organic solvents like toluene, chloroform and n-hexane with relative activities of 133%, 115.8% and 76.8%, respectively. *Pseudomonas aeruginosa* LST-03 lipase showed a similar trend of activity to the truncated AMS3 lipase. Overall, the native and truncated AMS3 lipases were organic solvents tolerant and stable at low temperatures. These features are suitable for enzyme applications in the synthesis of chiral intermediates at low temperatures.

## 3. Materials and Methods 

### 3.1. Predicted Structure and Validation of AMS3 Lipase (Native)

The structure of ASM3 lipase were predicted using Raptorx (raptorx.uchicago.edu) developed by the Xu group to predict protein structure and function. Predicted structure validations were performed using software such as SAVES (The Structure Analysis and Verification Server, UCLAS, MBI, Templeton, CA, USA) version 4 and RAMPAGE (http://www-cryst.bioc.cam.ac.uk/) for stereochemical parameters and Ramachandran plots. The software (SAVES, Los Angeles, CA, USA, version 4) analyzed ERRAT [44] and verified 3D [45], while the figures were prepared using the Chimera visual system (www.cgl.ucsf.edu/chimera).

### 3.2. Molecular Dynamic Simulation of Truncated AMS3 Lipase

The molecular dynamic simulation was carried out using Gromacs 4.0.5 [18]. The models were stimulated at 10, 20, 30 and 40 °C using constant pressure GROMOS 54a7 (www.gromos.net) force field. Total trajectory was 20 ns and snapshot was taken every 5 ns. The simulation allowed for better understanding of the dynamic properties of the enzyme in water at different temperatures. The root mean square deviation (RMSd) was computed for the protein backbone and residues in order to check the stability of the trajectories. Additionally, the root mean square fluctuation (RMSf) was computed per residue in order to study the flexibility of the trajectories.

### 3.3. Deletion of N-Terminal Domain of AMS3 Lipase 

#### Sub Cloning and Primer Design without N-Terminal Domain

A sub cloning strategy without an N-terminal domain was carried out. All cloning procedures were carried out as described by Sambrook (1987) [46]. The lipase gene was amplified using PCR with two primers designed according to restriction enzyme suitability of pET 51b(+)/ truncated AMS3 lipase (without the N-terminal domain) forward: 5′ GGTACCAGTTCCGCGTGGATC and reverse: 5′ GAGCTCTAGGCCGCAAGCT 3′. The truncated AMS3 lipase gene was isolated through PCR using 2 sets of primers mentioned above. The DNA was used as a template to perform Polymerase Chain Reaction (PCR) amplification of lipase gene. PCR amplification was carried using 25 µL reaction mixture that contained 12.5 µL of PCR mastermix, 1 µL (10 pmol) of primer forward and reverse, 2 µL of DNA template and 8.5 µL of distilled water on a thermocycler with a temperature program of pre-denaturation at 94 °C for 4 min, followed by 30 cycles of denaturation (94 °C, 1 min), annealing (60 °C, 1 min), extension (72 °C, 1 min) and finally extension at 72 °C for 7 min and preservation at 4 °C. The amplified product was electrophoresed on 1% (*w*/*v*) agarose gel with l Kb standard DNA marker at 70 mA for 35 min. The PCR product was visualized under UV light and purified using Combo kit (GeneAll, Seoul, Korea). The purified PCR product and the empty vector (pET 51b) were digested using Kpn1 and Sac1 restriction enzymes and ligated using T4 DNA ligase. The ligation mixture was used to transform competent cells of *E. coli* Top10 and incubated at 37 °C for 12 to 16 h. Positive transformants were selected based on clearing zones formation around the colonies. Positive transformants were grown in 10 mL of LB broth supplemented with ampicillin for 16 h and used for plasmid extraction. The plasmid containing the truncated AMS3 lipase was transformed into *E. coli* BL21 (De3) expression host, similar to the native.

### 3.4. Purification of Truncated AMS3 Lipase

Purification of truncated AMS3 lipase was conducted the same way with AMS3 lipase using affinity chromatography. The binding buffer was 100 mM Tris-HCl pH 8, 0.5 M sodium chloride and 20 mM Imidazole. The sample was eluted with 100 mM Tris-HCl pH 8, 0.5 M sodium chloride and 500 mM Imidazole. To increase protein homogeneity, the concentrated protein was purified again using gel filtration S200 (GE Healthcare, Chicago, IL, USA). The concentrated protein was placed into a XK 16 column containing resin S200 resin equilibrated with 100 mM Tris HCl pH 8.

### 3.5. SDS-PAGE and Native PAGE Analysis

SDS-PAGE was performed as described by Laemmli (1970) [47] using a discontinuous gel system; stacking (6%) and resolving (12%) in standard mini gel cassette (BioRad Mini Protean II, BioRad, Hercules, CA, USA). Tris-glycine running buffer pH 8.8 (25 mM of Tris, 192 mM of glycine and 0.1% (*w*/*v*) of SDS) was used for electrophoresis. Samples were mixed with 10× sample buffer (10% SDS, 20% glycerol, 0.05% Bromophenol Blue and 0.2 M of Tris-HCl pH 6.8) and loaded onto the gel. Electrophoresis was conducted at 200 V for 45 min. The gel was removed from the cassette and stained with staining solution (0.1% Coomassie Brilliant Blue R250, 50% methanol and 10% acetic acid) for 10–15 min. The gel was distained by washing several times with a distaining solution (10% (*v*/*v*) methanol and 10% acetic acid) to aid in visualizing the protein bands. Molecular size was determined through comparison with standard protein marker. The Native PAGE was conducted in a similar way to SDS-PAGE, except that SDS was eliminated during gel preparation and in both components of Tris-Glycine electrophoresis and loading buffers. Samples were prepared in non-denaturing condition (without boiling and addition of reducing agents such as β-mercaptoethanol and DTT) to allow the protein to exist in its native globular state.

### 3.6. Lipase Activity Assay

Assays were performed using the modified colorimetric method by Kwon and Rhee (1986) [48]. The reaction mixture was comprised of 1.0 mL of pure enzyme (enzyme and buffer), 2.5 mL of olive oil emulsion (50% olive oil + 50% phosphate buffer) and 0.02 mL of CaCl_2_. The reaction mixture was incubated at 5 °C under shaking at 200 rpm for 30 min. The reaction was terminated by adding 1.0 mL of HCl and 5.0 mL of isooctane. The upper layer (4.0 mL) was pipetted out into a test tube and added to a 1.0 mL of cupric acetate pyridine into the tube. Free fatty acids (FFA) dissolved in the isooctane were measured at an absorbance of 715 nm. Lipase activity was determined by measuring the amount of FFA. One unit of lipase activity was defined as the rate of 1 µmole of free fatty acids released in one minute.

### 3.7. Characterization of Truncated AMS3 Lipases

#### 3.7.1. Effect of Temperature and the Stability on Truncated AMS3 Lipase

Effect of temperature on purified truncated AMS3 lipases was measured at different temperatures ranging from 5 to 70 °C with an interval of 10 °C for 30 min. Lipase assay was performed colorimetrically at 200 rpm, using olive oil as a substrate. Enzyme stability test was conducted by first incubating the enzyme at various temperatures ranging from 5 to 70 °C for 30 min and assayed at optimum temperatures (50 and 60 °C). 

#### 3.7.2. Effect of pH and Stability on Truncated AMS3 Lipase

The effects of pH on the AMS3 lipase activity were measured at various pH values ranging from 4 to 12. The sample mixture (olive oil mix with different buffer ratio 1:1) was agitation at 200 rpm for 30 min. To study pH stability, the optimized buffer was mixed with olive oil in 1:1 ratio. The purified enzyme was pre-incubated at an optimum temperature in different range of buffers for 30 min and the activity was measured. The buffer systems used include the following: 50 mM acetate buffer (pH 4–6), potassium phosphate buffer (pH 6–8), Tris-Cl buffer (pH 8–9), glycine-NaOH (pH 9–11) and Na_2_HPO_3_/NaOH buffer (pH 11–12).

#### 3.7.3. Effect of Metal Ions on Truncated AMS3 Lipase

The effect of metal ions on lipase activity was studied using the method described by Kwon and Rhee (1986) [48] after the enzyme was pre-incubated at 60 °C for 30 min in 50 mM Tris-HCl buffer (pH 8.0) and either 1 mM or 5 mM of various metal ions in different reaction vessels Li^+^, Rb^+^, Na^+^, Mg^2+^, Ca^2+^, Fe^2+^, Mn^2+^, K^+^, Zn^2+^, Ni^2+^ or Co^2+^. Enzyme assay was performed at 60 °C using untreated enzyme as a negative control.

#### 3.7.4. Effect of Substrate Specificity on Truncated AMS3 Lipase Activity

Substrate specificity was determined using *p*-nitrophenol ester (acetate butyrate, deaconate, dodecanoate, tetradecanote, palmitate) in a reaction mixture that contained 2 µL of pure enzyme, 88 µL of 50 mM optimum buffer and 10 µL of substrate. The mixture was incubated at an optimum temperature of cold and heating block for 5 min. The reaction was terminated with ethanol. Free para-nitrophenol levels were determined using colorimetric lipase assay method. Absorbance was measured at 415 nm.

#### 3.7.5. Effect of Natural Oil on Truncated AMS3 Lipase

Natural oils (olive oil, corn oil, sun flower oil, canola oil, sesame oil, coconut oil and palm oil) were tested for the truncated AMS3 lipase activity. A lipase assay was performed colorimetrically at 60 °C for 30 min using the Kwon and Rhee (1986) method.

#### 3.7.6. Solvent Tolerant Profile of Truncated AMS3 Lipase

Organic solvent stability of truncated AMS3 lipase was carried out in a reaction mixture containing purified truncated AMS3 lipase with various solvents. The mixture containing 25% (*v*/*v*) of solvent and 50 mM of Tris-HCl buffer pH 8 was pre-incubated at 50 °C with shaking at 200 rpm for 30 min. Residual activity was measured as described by Kwon and Rhee (1986) using olive oil as a substrate. The solvents had varying log *p* values as follows: (1) DMSO (−1.45), (2) methanol (−0.76), (3) acetonitrile (−0.33), (4) ethanol (−0.24), (5) acetone (−0.24), propanol (0.28), (6) chloroform (2.0), (7) benzene (2.0), (8) toluene (2.5), (9) xylene (3.1) and (10) n-hexane (3.5).

#### 3.7.7. Predicted Structure and Validation of AMS3 Lipase (Native)

The structure of truncated AMS3 lipase was predicted using Raptorx (raptorx.uchicago.edu) by determining the protein structure and function developed by the Xu group. The predicted structure was validated in a manner similar to the method described in Section 3.1.

#### 3.7.8. Molecular Dynamic Simulation of Truncated AMS3 Lipase

The molecular dynamic simulation was carried out using Gromacs 4.0.5 [18]. The models were stimulated at 20 and 30 °C using constant pressure GROMOS 54a7 (www.gromos.net) force field. Total trajectory was 20 ns and a snapshot was taken every 5 ns. The simulation provided better understanding of the dynamic properties of the enzyme in water at different temperatures. The root mean square deviation (RMSd) was computed for the protein backbone and residues in order to check the stability of the trajectories. Additionally, root mean square fluctuation (RMSf) was computed for each residue to determine the flexibility of the trajectories.

## 4. Conclusions

The removal of the N-terminal domain of the AMS3 lipase was found to affect both the temperature and pH profile of the enzyme. The enzyme’s optimum temperature was 10 °C higher than the native lipase, whereas the pH stability profile changed from broad to more alkaline conditions. Computational molecular simulation of both lipases revealed certain structural changes which may contribute to these changes in biochemical properties. Generally, a reduction in unstable conformation via the deletion of the flexible region altered both the structure and pI values of the enzyme. These changes allow the modification of enzyme behaviors at different temperatures and pH levels. This study provides information for a rational design of lipase for academic and industrial application purposes.

## Figures and Tables

**Table 1:** Summary of N-terminal movement.

MD Simulation (Time)	Distance (Å)			
	Temperature			
	10 °C	20 °C	30 °C	40 °C
10 ns	33.55	17.17	14.84	18.91
15 ns	9.59	22.80	11.73	17.48
20 ns	12.84	14.13	4.53	4.91

**Table 2:** Truncated AMS3 lipase purification. The recombinant lipase was purified through one step affinity chromatography.

Step	Protein Concentration (mg/mL)	Total Protein (mg)	Lipase Activity (U/mL)	Total Activity (U)	Specific Activity (U/mg)	Fold	Recovery (%)
Crude	1.20	60	130.7	6535	108.9	1	100
Affinity	0.52	10.4	287.5	5750	552.8	5.07	87
Gel filtration	0.28	5.6	213.71	4274.2	763.25	7	65.4
